# Using deep learning for gene detection and classification in raw nanopore signals

**DOI:** 10.3389/fmicb.2022.942179

**Published:** 2022-09-15

**Authors:** Marketa Nykrynova, Roman Jakubicek, Vojtech Barton, Matej Bezdicek, Martina Lengerova, Helena Skutkova

**Affiliations:** ^1^Department of Biomedical Engineering, Faculty of Electrical Engineering and Communication, Brno University of Technology, Brno, Czechia; ^2^Department of Internal Medicine - Hematology and Oncology, University Hospital Brno, Brno, Czechia

**Keywords:** nanopore sequencing, squiggles, neural network, MLST, bacterial typing

## Abstract

Recently, nanopore sequencing has come to the fore as library preparation is rapid and simple, sequencing can be done almost anywhere, and longer reads are obtained than with next-generation sequencing. The main bottleneck still lies in data postprocessing which consists of basecalling, genome assembly, and localizing significant sequences, which is time consuming and computationally demanding, thus prolonging delivery of crucial results for clinical practice. Here, we present a neural network-based method capable of detecting and classifying specific genomic regions already in raw nanopore signals—squiggles. Therefore, the basecalling process can be omitted entirely as the raw signals of significant genes, or intergenic regions can be directly analyzed, or if the nucleotide sequences are required, the identified squiggles can be basecalled, preferably to others. The proposed neural network could be included directly in the sequencing run, allowing real-time squiggle processing.

## 1. Introduction

**Figure 1:**
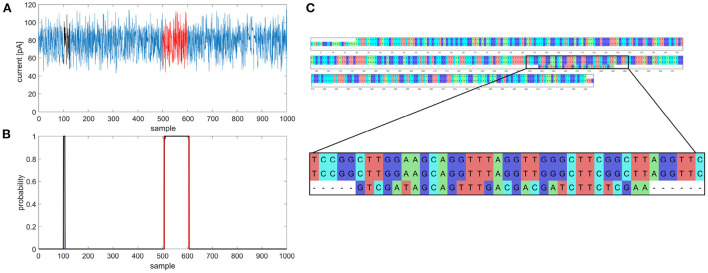
Example of multiple gene prediction, **(A)** squiggle with labeled positions of gene (red) and random match (black), **(B)** graph of predicted gene in squiggle, gene occurrence function is black, annotated position is red, **(C)** multiple sequence alignment of *gapA* template, *gapA* hit and random match.

DNA sequencing technologies revolutionized our ability to study genetic variations at the molecular level, which is necessary for a broad spectrum of applications from bacterial typing to cancer research. The introduction of the latest technology—nanopore sequencing—was a major breakthrough (Kono and Arakawa, 2019). Nanopore sequencing, unlike other methods, does not require DNA synthesis or amplification. Library preparation is significantly more straightforward, and sequencing can be performed practically anywhere (Hoenen et al., 2016; Castro-Wallace et al., 2017; Johnson et al., 2017). The technology uses small pores located in a membrane to which a voltage is applied. When the DNA strand passes through the pore, the electric current's changes are measured, and as a result, the current signal of the read called a “squiggle” is obtained. Each base in the squiggle is described by a different number of samples (measured values in time) as the speed of the strain passing through the pore is not constant. An example of a squiggle is shown in Figure 1A.

Besides the advantages of easy preparation and simple use, nanopore sequencing also provides reads with lengths from tens to hundreds of kilobase pairs (kbp), with a record of more than 2 Mbp (Amarasinghe et al., 2020). However, the main bottleneck in this technology lies in the low read accuracy, which is improving with each chemistry update, yet still does not compare with second-generation sequencers. Inaccuracies also emerged during the basecalling process, where the squiggles are converted to nucleotide sequences. The basecallers achieve an average read accuracy of 85–95% (Wang et al., 2021), which is insufficient for analyses such as single nucleotide variant detection. Although using high genome coverage in post-sequencing analysis, the consensus accuracy can be improved to 99.9% (Rang et al., 2018). If the basecalling process was bypassed and an analysis of the raw squiggles was performed, the results would be more precise and delivered in a shorter time as the Oxford Nanopore Technologies MinION sequencing platform allows real-time access to the sequencing run (Loose et al., 2016). Thus, waiting for the whole run to finish would not be necessary, and the crucial information could be analyzed almost in real-time. As the squiggle analysis can be performed during the sequencing itself, and the sequencing process can be stopped based on the real-time outputs from the analytical software, there is no need to use a flowcell's whole sequencing capacity.

As mentioned above, the basecalling process has lower read accuracy caused by several problems. Firstly, the change in current deviation does not correspond to one nucleotide passing through the nanopore, but approximately five nucleotides, leading to 4^5^ = 1,024 current levels (Lu et al., 2016). Moreover, the number of possible current levels can be higher because the 5-nucleotide step and the speed of the nucleotide strand passing through the nanopore are not constant. Secondly, the same bases can be chemically or epigenetically modified (e.g., 5-methylcytosine) (Wick et al., 2019), and the whole signal is also affected by noise. These complications mean that only neural networks (NNs) are used for basecalling nowadays (Wick et al., 2019). NNs have also found applications in other parts of nanopore sequencing, such as selective sequencing. An example of a tool used to determine whether to eject the sequenced molecule or continue sequencing could be SquiggleNet (Bao et al., 2021), which uses a convolutional neural network learned from the reference organism's sequencing data. The classifier then decides the sequenced segment's location and whether to continue sequencing or eject the molecule. Recently, NNs were also employed to distinguish mitochondrial DNA from genomic DNA in squiggles during a sequencing run (Danilevsky et al., 2022).

With NNs, it would be possible to identify specific genomic regions in raw nanopore data without the need for basecalling, as shown in the presented article. In particular, convolution layers are very useful for automatic extraction of relevant features in contrast to former approaches requiring manual feature extraction, selection or reduction, and subsequent classification. However, CNN-based features are indeed relevant for the intended purpose, but their abstractness is high, especially at higher network depths. In general, this is a problem for the possibility of correctly interpreting or understanding features related to the original input sequence. Such feature analysis is a long-standing problem and has been the research subject by some groups worldwide (like Bastidas et al., 2021). Nevertheless, for the purpose of simple gene detection and classification using a neural network, it is useless to understand intermediate features in the part of the network.

Using the proposed network, it is possible to find even more genes or gene fragments once the neural network can recognize and classify them. Unlike selective sequencing, the whole genomes could be sequenced, but crucial epidemiological information such as sequence type or presence of resistance genes can be obtained during the run. The rest of the sequencing data can then be processed for further analysis, such as core genome MLST. The proposed NN was used to predict and classify seven multilocus sequence typing (MLST) loci (*gapA, infB, mdh, pgi, phoE, rpoB, tonB*) in 29 *Klebsiella pneumoniae* genome squiggles. Raw squiggle analysis can bring more precise information, as the epidemiological and chemical modification can also be studied. In the future, distinguishing bacterial strains could be done using only the signals with no basecalling, providing crucial epidemiological information for early outbreak identification.

## 2. Materials and methods

### 2.1. Analyzed bacterium

*K. pneumoniae* is a Gram-negative opportunistic pathogen from the *Enterobacteriaceae* family. Usually, it affects immunocompromised patients, and the majority of *K. pneumoniae* infections are hospital-acquired. The gastrointestinal tract colonization generally occurs before nosocomial infections develop, which usually affect the urinary tract, respiratory tract or result in septicemia or soft tissue infection (Martin and Bachman, 2018; Choby et al., 2020). The genome size of *K. pneumoniae* is about 5.5 Mbp and incorporates about 5,000 to 6,000 genes from which 2,000 genes form the core genome, and almost 30,000 genes are parts of the pangenome (Wyres and Holt, 2016).

### 2.2. Genome sequencing

In this article, 29 *K. pneumoniae* isolates collected between 09/2014 and 07/2019, mainly at the Department of Internal Medicine, Hematology and Oncology at the University Hospital Brno, were analyzed. The high molecular weight DNA was extracted using the MagAttract HMW DNAKit (Qiagen, Venlo, NL), and the NanoDrop (Thermo Fisher Scientific, Waltham, MA, USA) was employed to measure the purity of the extracted DNA. The DNA concentration was checked by Qubit 3.0 Fluorometer (Thermo Fisher Scientific, Wilmington, DE, United States) and using Agilent 4200 TapeStation (Agilent Technologies, Santa Clara, CA, USA) the proper length of the isolated DNA was checked. The Rapid Barcoding Kit (Oxford Nanopore Technologies, Oxford, UK) was used to prepare the sequencing library for 27 *K. pneumoniae* isolates. For the remaining two isolates, the Ligation Sequencing 1D Kit (Oxford Nanopore Technologies, Oxford, UK) was used to prepare the library for Oxford Nanopore sequencing. The sequencing was performed using the MinION sequencing platform (Oxford Nanopore Technologies, Oxford, UK) with R9.4.1 flowcells.

The sequenced genomes were basecalled and, in the case of the pooled library, separated according to barcodes using Guppy software (3.4.4+a296acb). The data quality was checked using PycoQC (v2.2.3, Leger and Leonardi, 2019). See Supplementary Table 1 for detailed information about each sequencing run. The analyzed datasets can be found in the National Center for Biotechnology Information Sequence Read Archive database under a BioProject with accession number PRJNA786743.

### 2.3. Neural network architecture

To sort out the task of enabling gene localization and classification, some challenges had to be overcome. The main problem was squiggles that were too long, moreover with uneven sampling time. This distinctly increases computational complexity and memory demands, and first and foremost, causes a significant vanishing gradient (VG) effect during training, especially for the recurrent nets. Further, conventional neural networks cannot be applied to signals of different lengths, and they require strictly the same input length.

**Figure 2:**
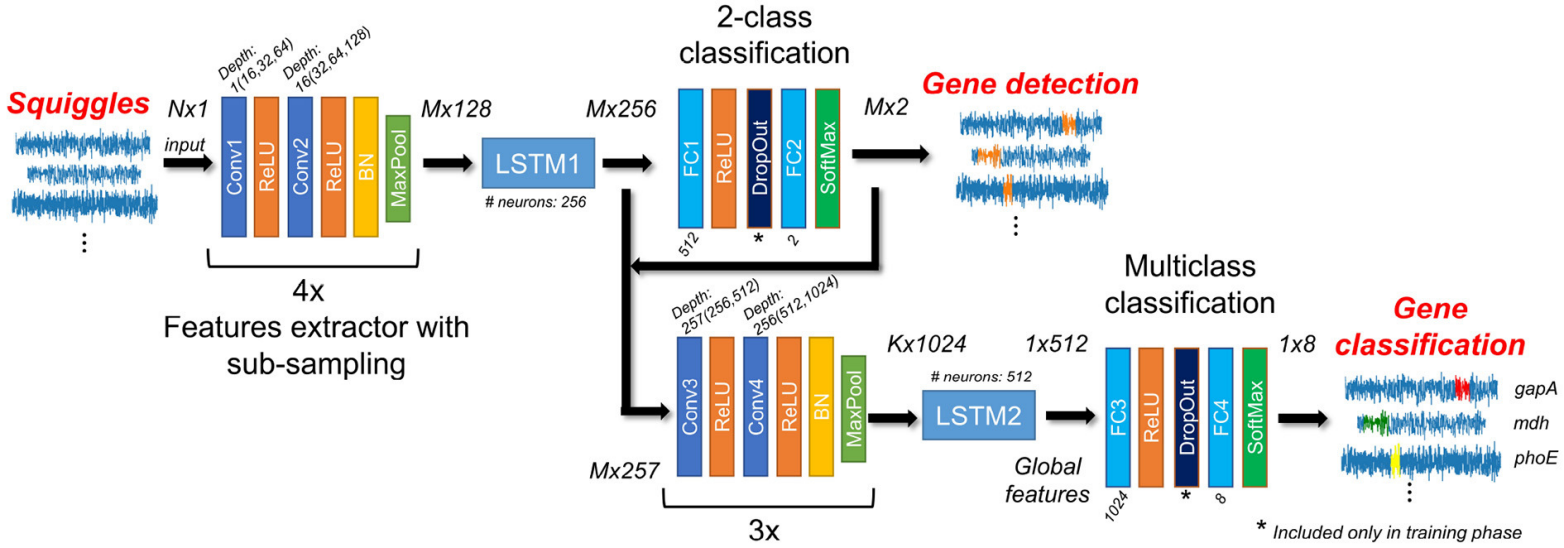
Architecture of NanoGeneNet. Each encoder block contains two convolution layers (Conv) with a kernel size equal to 3, followed by ReLU activation function and finally a Batch Normalization (BN) and a Maxpooling layer with size kernel and stride of 2. Network also includes two Long Short-Term Memory (LSTM) recurrent networks and classification blocks composed of Fully Connected layers (FC), SoftMax activation layers and a DropOut layer used only in the training phase. For each block, tensor size shown in the form of *squiggle length x feature number*, where *N* is original squiggle length, *M* is its downsampled version length after first encoder and *K* is length after second one.

The proposed NanoGeneNet can be divided into three basic parts: feature extraction, gene localization (so-called a sequence-to-sequence regime) and finally, gene classification with another feature extraction (a sequence-to-vector regime). Using the combination of convolution and recurrent networks turned out to be a great solution for long and uneven signal lengths. For more details on architecture design, see Figure 2.

The network was realized in Python 3.9 with the PyTorch library. The source code for training, validation, and especially feedforward for NanoGeneNet is available on GitHub along with a demo and an example squiggle.

#### 2.3.1. Local feature extraction with downsampling

The encoder part of the net allows local features to be extracted from the raw signal using a recursive repeating block. As shown in Figure 2, the one block contains two convolution layers (Conv) with a kernel size of 3 and depth of 16, doubled in each recursion. The Conv layer is always followed by a ReLU activation function, and the block ends with a batch normalization layer and non-linear spatial reduction layer—MaxPooling [2x2;2]. In the encoder part, the repeating blocks are connected in series. This encoder part occurs twice in NanoGeneNet; the first time with four blocks and the second time with three blocks.

The network part designed in this way provides a new local feature signal reflecting the occurring or non-occurring gene from an input signal (squiggle) with length *N*. Due to the MaxPooling layer, there is a spatial reduction in the signal length of *M* with a sub-sampling factor of 2^4^ to decrease computational complexity, while the only relevant features for gene detection are retained during net feedforward. Since this part of the network only views a very local part of the signal, the following LSTM (Long Short-Term Memory) (Hochreiter and Schmidhuber, 1997) network has the task of viewing the whole signal globally and determining the local features for each signal sample, considering all previous samples (long memory). The output is then the tensor of local feature signals sized *M*× 256, where *M* is the length of the downsampled feature signals.

Due to a recurrent LSTM net, the input can be different length signals and sub-sampling mildly suppresses the VG effect and significantly decreases GPU memory demands. The complete suppression of VG problem is performed by multistage training.

#### 2.3.2. Gene occurrence prediction

**Figure 3:**
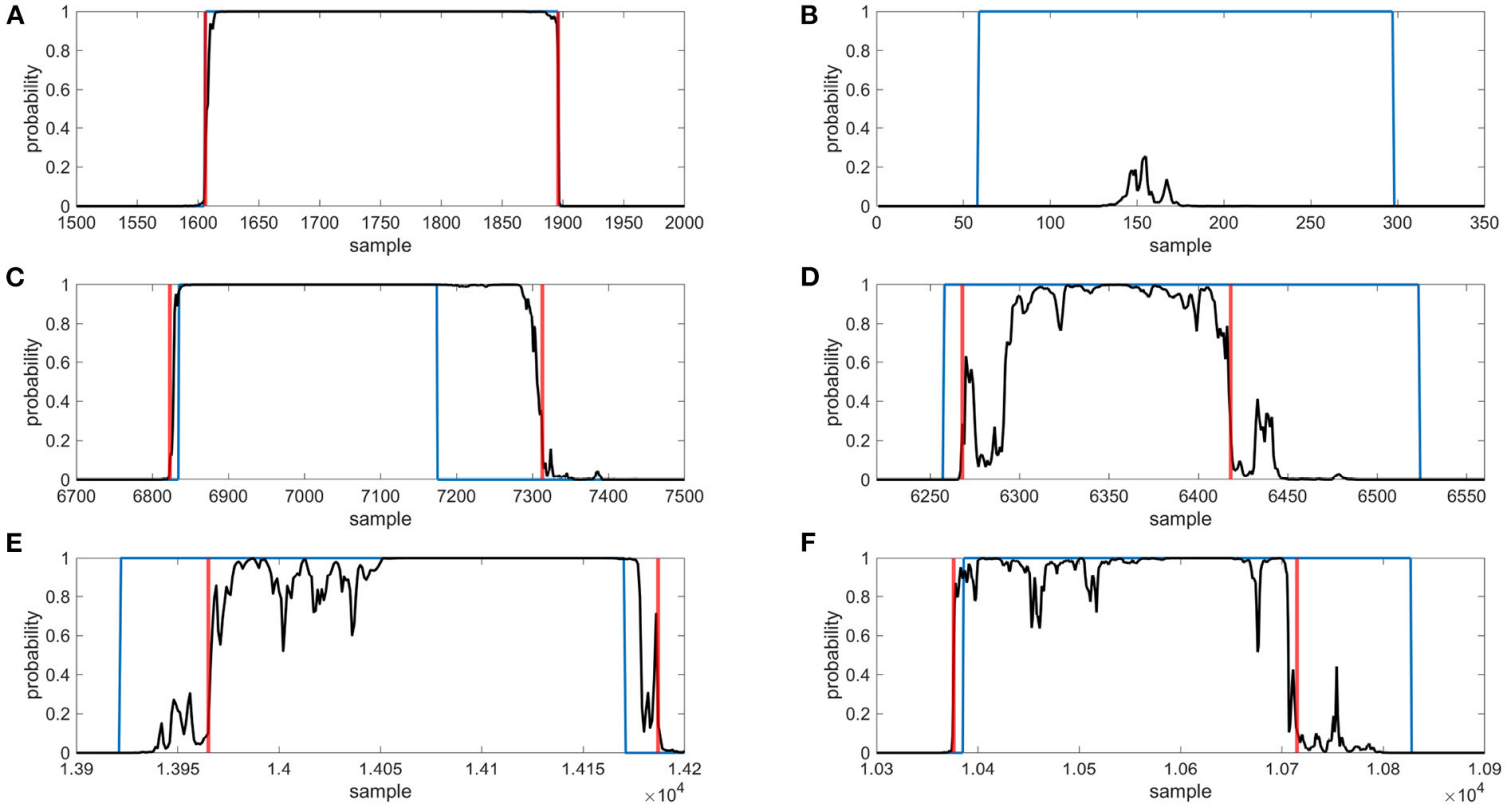
Examples of gene detection, annotated positions are blue, gene occurrence functions are black and detected gene positions are red, **(A)** gene was found correctly, **(B)** gene was not found, **(C)** gene was outside annotated borders, **(D)** gene was inside annotated borders, **(E)** gene was shifted to right, **(F)** gene was shifted to left.

From the previous LSTM block (LSTM1), each sample of the output tensor of length *M* (corresponding to downsampled squiggle) is now encoded with a vector of 256 features. Its depth is only one, thus it uses only one LSTM network containing 256 hidden neurons. The output tensor subsequently inputs the two-class classification part. Here, two fully connected (FC) networks with ReLU and Softmax activation function can be used, respectively. The first FC layer (FC1) contains 512 neurons and the second layer FC2 contains only two neurons. This part of the proposed NanoGeneNet classifies each downsampled signal sample into two classes; gene or non-gene. The output is tensor *M*× 2 defining probability assignment to all classes. Since there are two classes, we can use the probabilities of only the first class as an output gene occurrence function. Examples of such functions are shown in Figure 3.

#### 2.3.3. Gene classification

In this part, the local feature tensor from the previous encoder and LSTM is concatenated with the obtained likelihood function and the resulting tensor is *M*× 257 in size. The tensor is encoded into a new feature tensor of length *K* corresponding with the feature import to classify gene types via a second encoder block with a sub-sampling factor of 2^3^ (second encoder). Then using another LSTM network (LSTM2) with depth one and containing 512 neurons, the new local feature tensor is transformed into another new tensor (1 × 512) reflecting only the whole signal's global features to enable classification of the whole signal into gene class. This task is performed via the last part of NanoGeneNet—the multiclass classification network. It has the same architecture as the previous 2-class net, except that the last FC layer (FC4) has eight neurons followed by SoftMax, enabling a one-hot coding of output class probabilities. The final decision is based on the choice of class with the maximal value.

### 2.4. Network training details

#### 2.4.1. Training and validation dataset

The available signal dataset was divided on the genome level by hand to ensure data distribution consistency for training and validation. For the validation dataset of each gene, all randomly selected genome signals were selected to make up something between 20 and 30% of the entire database. It cannot be done exactly because each file contains a different number of signals for specific genes within each genome. As the genome includes the same gene sequence, all signals always within the genome were used for training/testing; ignoring the uneven time sampling, i.e., a non-linear scaling, which is taken as a kind of augmentation useful for training.

#### 2.4.2. Multistage approach

Generally, the VG problem is related to the signal length, the longer the signal, the greater the effect of this problem. Therefore, multistage training was proposed. In the first phase, the first encoder with sub-sampling was pre-trained on shorter signals. In each iteration, the current signal was randomly cut with random termination and random length in a range of 20-80 thousand samples. In the case of gene signal occurrence, this cut training signal always contained the whole gene. In this way, the encoder and LSTM1 training were achieved with a minimal VG problem. To derive a loss function during training, the two-class classification was trained concurrently based on cut annotations in this way.

In the second phase, the first encoder was additionally retrained on the database containing the non-gene signals. Finally, in this way, the pre-trained net (encoder, LSTM1 and FC two-class net) was fine-tuned for signals with genuine length. The same training strategy was also used for the second part of NanoGeneNet, but in this case for an eight-class whole-signal classification task and with its first part frozen.

#### 2.4.3. Training parameters

All training dataset signals were randomly shuffled within each new iteration, and all nets were trained from scratch. As both tasks were defined as a classification with a class probability estimation, a weighted cross-entropy was chosen as a loss function. Further, an ADAM algorithm (Adaptive Moment Estimation) (Kingma and Ba, 2014) was used as an optimization algorithm, and the initialization Learning Rate (LR) was set to 0.001, and the weight decay to 0.0001. During all stages of training, the LR was manually changed (decreased) based on the designer's experiences. A batch size greater than one could only be used in the first training stage, where the net worked with cut signals; here, the batch size was 8, and a larger batch size resulted in too much regularization. In the training phase, the Dropout layer (Srivastava et al., 2014) worked with a probability of 50%. Other hyperparameters were set by default in PyTorch, which can be found in the shared source code on GitHub.

#### 2.4.4. Hardware requirements

NanoGeneNet training was performed on a computational device with Intel Xeon E5-2603v4, 16 GB RAM and nVidia Titan Xp, 12 GB GDDR5 graphics card. The network was realized in Python 3.9 with the PyTorch library. One stage of training took around 2 h, and required up to 10 epochs for a sufficient training determined by an already non-decreasing loss function of the training and validation set under expert control.

The minimal hardware requirements depended on the input squiggle length, where the above-mentioned device had sufficient parameters to process our database. The prediction and classification of a squiggle with median of length (143 thousand samples) took around 0.2 s in total. There is a linear dependence between the signal length and computational time, where the longest squiggle from our database (almost 4.5 million samples) took around 4 s.

### 2.5. Gene coordinates detection in predicted signals

The outputs from the first part were gene occurrence functions determining the probability for each sample in the downsampling signal being part of the gene. The positions of the predicted gene were detected to evaluate the prediction accuracy. In each gene occurrence curve, the peaks exceeding a threshold of 0.9 were found and further analyzed as possible target genes. The shortest possible distance between two peaks was set to 100 samples; otherwise, the peaks were merged into one. Boundaries where the gene occurrence curve exceeded 0.5 were located around possible gene peaks in the next step. Then the gene boundaries were extended sample by sample to find a precise prediction beginning and end. The samples were added to the gene until the difference between the two adjacent samples was higher than 0.1.

For each calculated gene's coordinates, the dice coefficient was calculated as


(1) $$\begin{array}{r} DSC=\frac{2TP}{\left(2TP+FP+FN\right)}, \end{array}$$

where *TP* was the number of samples correctly classified as gene, the *FP* was the number of samples falsely labeled as gene, and *FN* was the number of samples falsely labeled as not-gene. For examples of dice calculation, including determination of TP, FP, and FN samples, see Supplementary Figure 1. If just one peak was observed in the signal, the dice was calculated for it. In cases where more peaks were detected in the gene occurrence function, the coefficient was calculated for each of them. For gene prediction and detection evaluation, the detected gene positions with the highest dice were selected.

## 3. Results

### 3.1. Analyzed genes

The seven MLST loci (*gapA, infB, mdh, pgi, phoE, rpoB, tonB*) from BIGSdb (Jolley and Maiden, 2010) were chosen for showing the proposed neural network's performance. The first allele from each MLST gene was searched for in basecalled reads. The median lengths of the located MLST genes were, on average, 4 bp shorter than the queries. Thus, most of the identified genes contained almost the whole allele sequence. The analyzed sequence variability was about 6.31% (Table 1). This variability is caused mainly by the presence of other alleles from a given gene in the dataset, as only one allele for each gene was searched for. Based on these values, it can be said that the dataset is variable enough to train the network to be able to recognize different gene's alleles.

**Table 1:** Statistics about seven analyzed MLST loci.

	**Query allele** **length [bp]**	**Number of alelles ** **in dataset**	**Identified gene ** **median length [bp]**	**Variability ** **of identified gene [%]**
*gapA*	450	4	446	6.35
*infB*	318	6	314	5.57
*mdh*	477	5	471	6.14
*pgi*	432	7	428	6.32
*phoE*	420	11	415	6.59
*rpoB*	501	7	499	5.81
*tonB*	414	14	409	7.40

### 3.2. Dataset preparation

Gene signals that could be used to train the neural network and later validate its performance were obtained by the following described process. BLAST (2.9.0+, Camacho et al., 2009) was employed to localize sequences of interest. The fast5 files containing basecalled fastq sequences were used to prepare the signal database. Then, the gene templates were examined in basecalled data, and results (hits) were saved in CSV format. In addition, the whole squiggles containing the gene sequences were extracted and saved in an internal h5 format, so they could be swiftly accessible and easily modified.

If more genomes were sequenced in one run, the BLAST results contained hits for all genomes from a particular run. To assign barcodes to the hits, demultiplexing tables were created for sequencing runs where barcoding kits were used. The outputs from the Guppy barcoder were used for this purpose. Each created table contained all reads ID from the sequencing run and their corresponding barcodes; so, it was possible to add sample identification to the hits in the BLAST table.

The BLAST results were filtered to remove random and partial hits. For further processing, only the hits with a percentage of identical matches more significant or equal to 90%, the length at least 90% of query length, and the e-value lower or equal to 1e-50 were chosen. The searched sequences were found on both the leading and complementary strands.

In the last signal extraction step, the gene sequences' BLAST coordinates were recalculated to signal coordinates. Thus, a dataset containing squiggles with desired genes was created. To each squiggle, the gene signal coordinates were added. The neural network should also recognize squiggles without genes; therefore, datasets with no genes were created.

For preprocessing the raw sequencing fast5 files and dataset preparation, the internally developed MANASIG (Barton et al., 2021) package was used and is available on GitHub.

### 3.3. Signals for training/validating neural network

In total, 48,860 squiggles from which 38,867 contained one of the seven housekeeping genes fragments were analyzed. The length of squiggles with genes ranged from 4,362 to 4,477,607 samples with a median of about 142,869 samples. The median gene fragment lengths were 4,815 (*gapA*), 3,417 (*infB*), 5,197 (*mdh*), 4,643 (*pgi*), 4,586 (*phoE*), 5,387 (*rpoB*), and 4,565 (*tonB*) samples. The number of signals with no genes was 9,993 and their lengths varied from 2,008 to 1,411,573 samples with a median value of 24,408 samples. These squiggles were randomly generated from the seven sequencing runs. For a detailed number of squiggles from each analyzed genome, see Supplementary Table 2.

For neural network training, about 75% of all squiggles were used, and the rest were used to validate NN performance.

### 3.4. Gene detection in squiggles

From the validation dataset, 41 squiggles with corrupted gene coordinates, where the metadata and raw data did not match, were removed. In total, 11,887 squiggles were used for validation, of which 2,000 had no gene sequences. The representation of individual genes in the dataset was as follows: *gapA*—1,671, *infB*—1,570, *mdh*—1,644, *pgi*—1,659, *phoE*—1,160, *rpoB*—1,179, and *tonB*—1,004.

The gene predictions in the downsampled squiggles containing any genes of interest were classified into two categories—a gene found, and a gene not found. To evaluate, the calculated dice coefficients were used. On average, the detection success rate was about 98%; see Table 2 for specific values. The gene found category was further split into the other two subgroups—the gene was found correctly (dice ≥ 0.9), or the gene was shifted. If the predicted gene was labeled as shifted, four variants could be observed—the detected part was inside/outside the annotated borders or shifted before/beyond the start/end of the gene. Examples of all possible gene detection cases are shown in Figure 3, and squiggle percentages in the given categories can be seen in Table 3. The prediction with dice greater than or equal to 0.9 differed from the annotation position by, on average, about ten samples. Predictions with lower dice consisted mainly of genes shifted beyond the annotated gene start and end, and from predictions inside annotated gene boundaries.

**Table 2:** Percentages of identified or non-identified MLST loci.

**[%]**	***gapA***	***infB***	***mdh***	***pgi***	***phoE***	***rpoB***	***tonB***
Gene found	99.04	98.41	97.51	96.08	98.88	98.98	98.61
Gene not found	0.96	1.59	2.49	3.92	1.12	1.02	1.39

**Table 3:** Percentages of identified MLST loci included shifted genes.

**[%]**	***gapA***	***infB***	***mdh***	***pgi***	***phoE***	***rpoB***	***tonB***
Gene was found correctly	95.75	93.50	94.71	90.84	93.71	95.17	94.62
Gene was shifted	3.29	4.90	2.80	5.24	5.18	3.82	3.99
- Outside annotated borders	0.06	0.25	0.18	0.48	0.09	0.17	0.60
- Inside annotated borders	1.38	1.34	1.58	2.29	1.81	1.27	1.20
- Shifted beyond gene start/end	1.68	3.31	0.97	2.35	3.10	2.29	2.19
- Shifted before gene start/end	0.18	0.00	0.06	0.12	0.17	0.08	0.00

In the case of squiggles with no genes, predictions with dice equal to one were marked as correct. In total, 96.6% of squiggles without any genes were successfully recognized, and no gene was detected in them.

### 3.5. Gene squiggles classification

The squiggles from the validation dataset were classified via the proposed neural network into eight categories, where seven categories were for analyzed genes, and the last one was for the squiggles with no gene. For each squiggle, the probabilities that the gene belongs to a given category were established. From these values, the maximum was chosen, and the squiggles were assigned to a given category.

**Figure 4:**
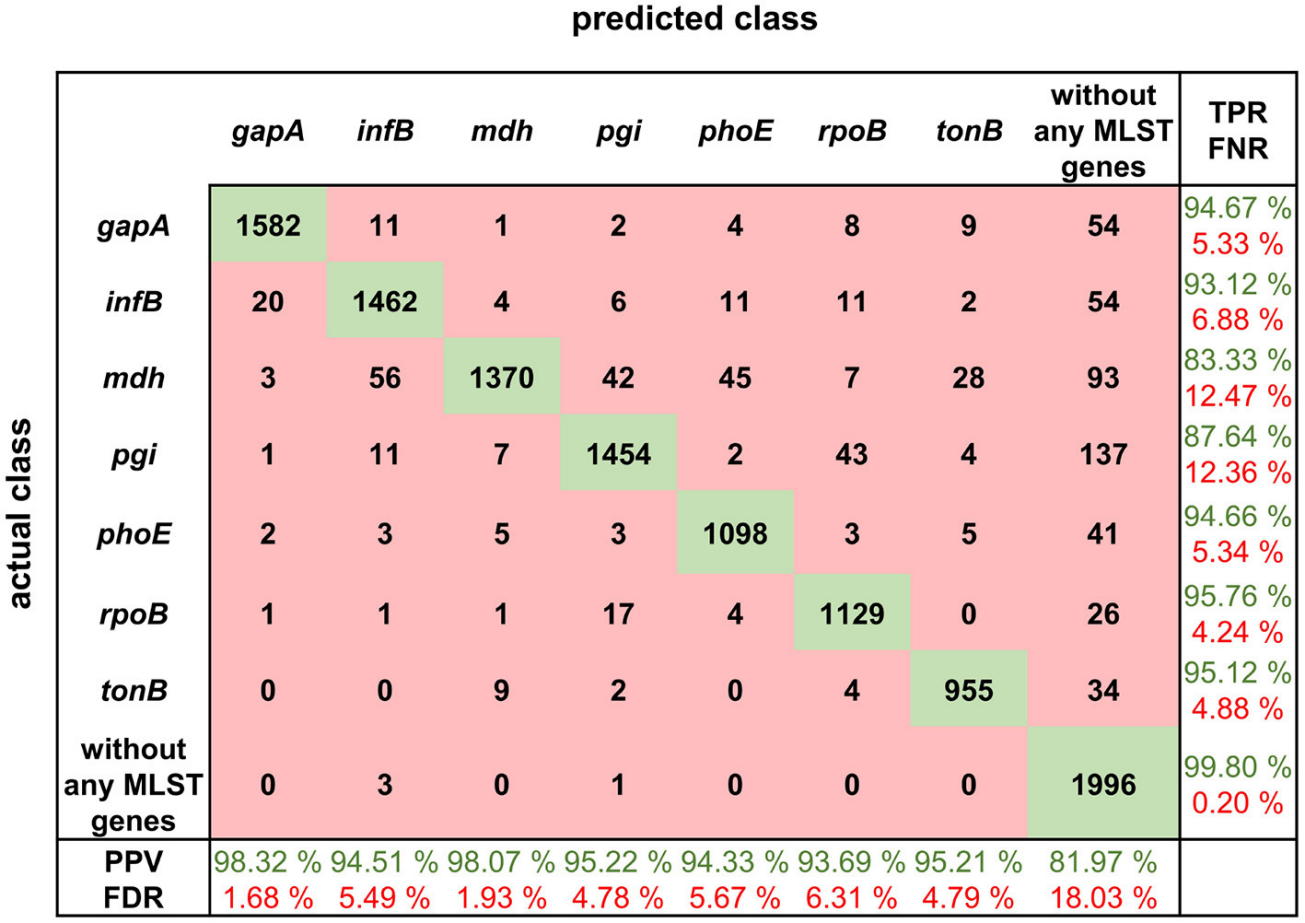
Confusion matrix for MLST loci classification results with calculated true positive rates (TPR), false negative rates (FNR), positive predictive values (PPV), and false discovery rates (FDR).

The squiggles with no MLST genes were correctly classified in 99.80% of cases and only four squiggles were misclassified. The true positive rate (TPR) of gene classification was about 94.67% for five out of seven MLST loci. In the case of *pgi*, the TPR dropped to 87.64% and in the case of *mdh* to 83.33%. See the detailed results in Figure 4.

The confusion matrix shows the true positive rate, which is the highest for the squiggles with no detectable gene and in the case of MLST genes, the highest value of 95.76% is for *rpoB* gene. In general, the normalized true positive value for all MLST genes was 92.04%; if squiggles without genes are included, the average true positive rate is even higher, at 93.01%.

On the other hand, the false discovery rate (FDR) was the highest for the squiggles without genes. The difference between the average false discovery rate for the squiggles with and without genes is 13.65%. It can be concluded that there is a much higher rate of not identifying the gene than identifying it incorrectly.

## 4. Discussion

**Figure 5:**
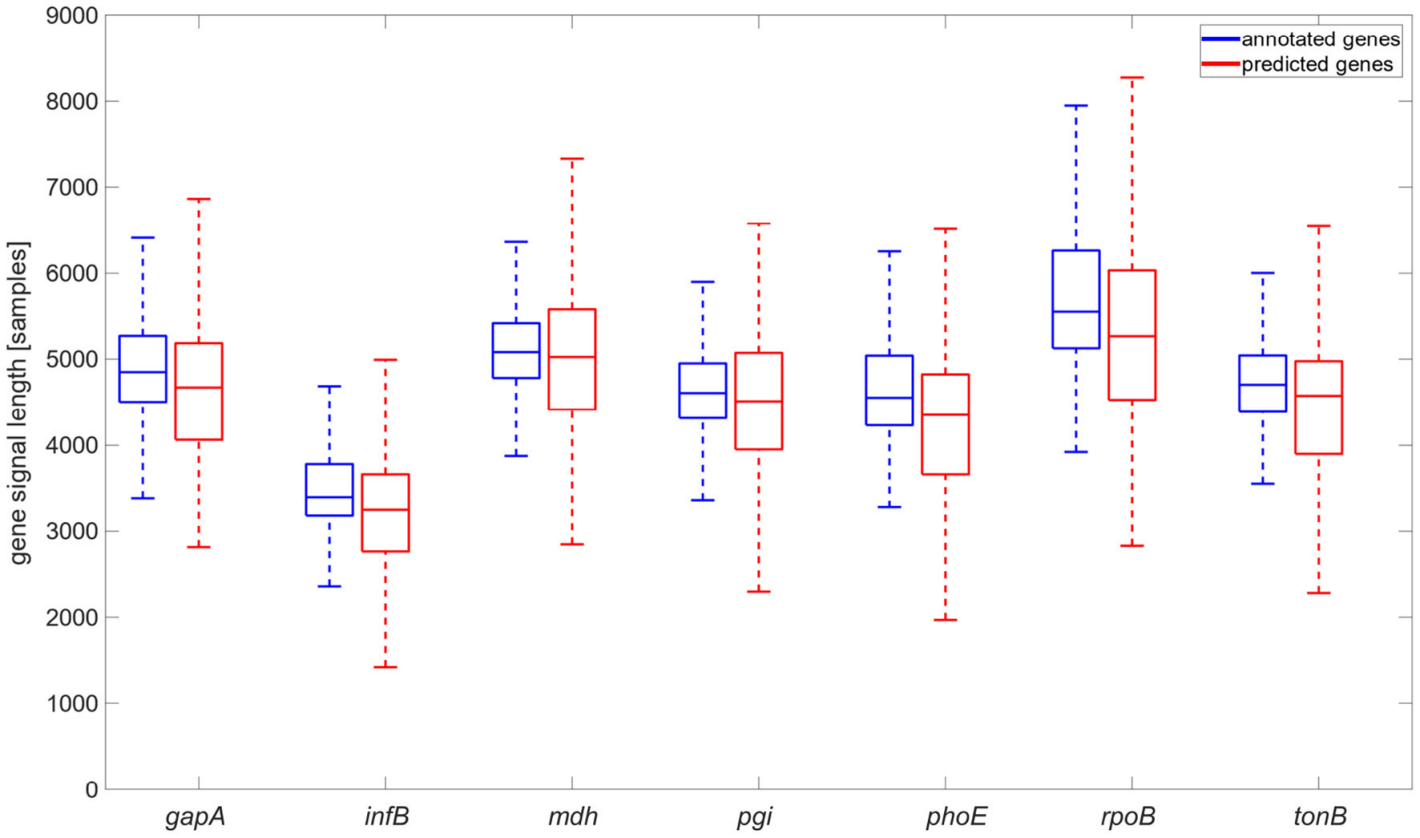
Comparison of annotated gene signal length and gene signal length identified by the neural network, graph is shown without flyers for clearness.

A comparison of detected and annotated gene coordinates (see Figure 5) and their overlaps was conducted. The results showed that they came from the same statistical distribution. The neural network tended to identify the genes a bit longer than annotated. This phenomenon can be caused by the signal length irregularity in the same sequence. Also, the signals were downsampled for the neural network. This pre-processing and the need to recompute the positions of genes to the original space signal can cause some variations in the genes' signals lengths.

The sequencing data are stored in the fast5 format, which is based on hdf5 format. However, hdf5 is not fully backwards compatible; the hierarchical structure can change with every new nanopore chemistry or software upgrade, and no complex descriptions about the fast5 nanopore format and its modification exist. For this reason, our proposed package for fast5 file processing can be used with R9.4.1 flowcells, two sequencing kits (SQK-RBK004, SQK-LSK109) and nanopore MinKNOW v19 and v20 sequencing software; with other versions, it might be necessary to modify the package. Completely new chemistry can also influence signal properties such as squiggle lengths, and in that case, new neural network training would be needed. Also, the neural network may not work correctly if the squiggle lengths in the training/validating dataset contain outliers such as extremely long ones.

During the gene coordinates' detection in predicted signals, it was found that gene occurrence functions sometimes contained more than just one peak exceeding a specified threshold, as shown in Figures 1A,B. Multiple predictions were observed in 3.81% squiggles from the validation dataset, and in the majority of cases (more than 90%), two peaks were predicted. After detecting peak coordinates, the corresponding basecalled nucleotide sequences were analyzed. It was found out that the false positive predictions contained sequences with a partial match to the desired gene, see Figure 1C. Nevertheless, the partial matches were significantly shorter than the examined sequences; therefore, they could be filtered in postprocessing. However, there could be a problem with setting the filter parameters because the partial matches may have a different numbers of samples in the squiggles as the speed of DNA passing through the pore is not constant and the sampling is non-equidistant. This mentioned multiple detection problem could be solved if the genes or other specific sequences we wanted to search for are unique and non-repetitive in the analyzed bacterial species. On the other hand, from multiple detection results, it can also be said that the neural network could find even short signals that corresponded to several dozen base pair long sequences, such as parts of genes. In addition, it could recognize the squiggles even if there were many mutations; thus, the network can be used to predict and classify even highly variable genomic regions.

If more than one gene is located in the analyzed squiggle, it could cause a problem for proper neural network function. Multiple gene presence may occur if long squiggles are produced during the sequencing process. To avoid this problem, genomic regions we wanted to detect should be carefully selected. The first option is to choose genes that have sufficient distance from each other in the analyzed species. The second option is to pre-process the squiggles before sending them into a neural network. Signals longer than a given threshold can be divided into several shorter signals, ensuring that only one targeted region will be present in each signal.

In a future project phase, it will be desirable to perform a more detailed analysis of the proposed model's behavior on newly acquired real squiggles. Using time-consuming experiments, it will be possible to observe which parts of the squiggles are crucial for gene localization or classification related to the biological nature of these signal parts. In addition, other more sophisticated tools for analyzing machine learning models, e.g., (Ancona et al., 2018), can be used. Based on the initial insights gained during the model design and learning with augmentation, it can be concluded that, for example, for gene localization, the unidirectional LSTM network is strongly dependent on the presence of the initial part of the gene sequence being searched. Experimentally, it has been found that after replacing the entire gene part with a randomly selected non-gene signal, the network is still able to localize the gene successfully but, naturally, does not classify it correctly. Thus, the network uses complex information, especially from the part immediately before the gene segment, and the information in the region of the searched gene is irrelevant. On the other hand, the information in the localized gene part is crucial for classification due to the proposed architecture.

## 5. Conclusion

This paper presented a deep learning method to identify and classify specific genomic regions in raw nanopore sequencing data. The proposed neural network can be used to find whole genes, their parts or intergenic regions. We showed one of the possible neural network uses—detecting and classifying seven MLST loci in *K. pneumoniae* genomes in squiggles.

The percentage of correctly predicted genes was 98.2%, and they were successfully classified in 92.9% cases. The squiggles with no MLST loci were correctly predicted in 96.6% and classified in 99.8% cases. The NN achieved the same accuracy as basecalling tools, and if postprocessing was employed, the accuracy could be even higher.

The main requirement of the proposed approach for gene prediction and classification is a large amount of data to train the neural network. Without sufficient data, the network would not be adequately trained, and precise results could not be obtained. From the study, it can also be said that the genomic regions detected via the neural network should be unique, non-repetitive sequences of any length and should be located in the analyzed genomes with sufficient distances between them.

Nanopore sequencing has huge potential in routine clinical practice. It can be used instead of time consuming NGS to deliver crucial epidemiology information earlier. For example, if there is a need to analyse samples from one hospital department to find out if there is an outbreak or not, if an infection is spreading via instruments, patients or medical staff, nanopore sequencing can be employed. NGS analysis would consist of library preparation, sequencing, and *in silico* sequence type determination from post-processed sequencing data, which can take 2–3 days while the potential epidemic spreads uncontrollably. The results could be delivered the same morning if nanopore sequencing combined with the proposed NN is employed. The squiggles containing MLST loci can be recognized during a run or immediately after it and analyzed. If more genomes are sequenced in one run, the barcodes attached to MLST squiggles can be used to find out which, e.g., *gapA* loci belongs to which genome.

The network can be trained to recognize different significant sequences. Hence, the proposed approach can be used for other purposes, such as direct clinical sample sequencing. Thus, cultivation is not needed, which significantly shortens the time for obtaining the results. The network can be trained for various analyses such as filtering out the human sequences, identifying MLST loci and determining infectious agents' sequence types, spa-typing of *Staphylococcus aureus* or K typing of *K. pneumoniae* strains, and other bacterial strain typing schemes. Moreover, phenotype prediction and, therefore, targeted administration of antimicrobials can be made in a very short time due to the absence of the basecalling step. Microbial species identification is also possible using the network with an appropriate squiggle database.

## Data availability statement

The datasets presented in this study can be found in online repositories. The names of the repository/repositories and accession number(s) can be found below: https://www.ncbi.nlm.nih.gov/, PRJNA786743.

## Author contributions

MN, VB, and HS contributed to the conception and design of the study. MN and VB created and implemented the algorithm for fast5 processing and evaluated the results. RJ designed and implemented the neural network. ML and MB ensured the biological aspects of the project. MN and RJ wrote the manuscript. All authors read and approved the final manuscript.

## Funding

This work has been supported by grant FEKT-K-21-6912 realized within the project Quality Internal Grants of BUT (KInG BUT), Reg. No. CZ.02.2.69/0.0/0.0/19_073/0016948, which is financed from the OP RDE. Collecting, processing, storing and sequencing of all bacterial isolates used in this study was supported by the Ministry of Health of the Czech Republic, Grant No. NV19-09-00430, all rights reserved.

## Conflict of interest

The authors declare that the research was conducted in the absence of any commercial or financial relationships that could be construed as a potential conflict of interest.

## Publisher's note

All claims expressed in this article are solely those of the authors and do not necessarily represent those of their affiliated organizations, or those of the publisher, the editors and the reviewers. Any product that may be evaluated in this article, or claim that may be made by its manufacturer, is not guaranteed or endorsed by the publisher.

## Supplementary material

Supplementary Figure 1 Examples of dice coefficient calculation.

Supplementary Table 1 Sequencing runs information.

Supplementary Table 2 Number of squiggles containing given MLST locus for each analyzed genome and number of squiggles with no MLST genes for given sequencing run.
